# A Piezoresistive Array Armband With Reduced Number of Sensors for Hand Gesture Recognition

**DOI:** 10.3389/fnbot.2019.00114

**Published:** 2020-01-17

**Authors:** Daniele Esposito, Emilio Andreozzi, Gaetano D. Gargiulo, Antonio Fratini, Giovanni D’Addio, Ganesh R. Naik, Paolo Bifulco

**Affiliations:** ^1^Department of Electrical Engineering and Information Technologies, Polytechnic and Basic Sciences School, University of Naples Federico II, Naples, Italy; ^2^Department of Neurorehabilitation, IRCCS Istituti Clinici Scientifici Maugeri, Pavia, Italy; ^3^School of Computing, Engineering and Mathematics, Western Sydney University, Penrith, NSW, Australia; ^4^School of Life and Health Sciences, Aston University, Birmingham, United Kingdom; ^5^MARCS Institute for Brain, Behaviour and Development, Western Sydney University, Penrith, NSW, Australia

**Keywords:** muscle sensors array, piezoresistive sensor, human–machine interface, hand gesture recognition, support vector machine, exergaming

## Abstract

Human machine interfaces (HMIs) are employed in a broad range of applications, spanning from assistive devices for disability to remote manipulation and gaming controllers. In this study, a new piezoresistive sensors array armband is proposed for hand gesture recognition. The armband encloses only three sensors targeting specific forearm muscles, with the aim to discriminate eight hand movements. Each sensor is made by a force-sensitive resistor (FSR) with a dedicated mechanical coupler and is designed to sense muscle swelling during contraction. The armband is designed to be easily wearable and adjustable for any user and was tested on 10 volunteers. Hand gestures are classified by means of different machine learning algorithms, and classification performances are assessed applying both, the 10-fold and leave-one-out cross-validations. A linear support vector machine provided 96% mean accuracy across all participants. Ultimately, this classifier was implemented on an Arduino platform and allowed successful control for videogames in real-time. The low power consumption together with the high level of accuracy suggests the potential of this device for exergames commonly employed for neuromotor rehabilitation. The reduced number of sensors makes this HMI also suitable for hand-prosthesis control.

## Introduction

Human machine interfaces (HMIs) are becoming increasingly widespread with applications spanning from assistive devices for disability, muscle rehabilitation, prosthesis control, remote manipulation, and gaming controllers (McKirahan and Guccione, 2016; Boy, 2017; Beckerle et al., 2018). Being the hand extremely important in one’s life, an entire field of HMI is dedicated to hand gesture recognition applications (Arapi et al., 2018; Shukla et al., 2018). Generally, visual, electromyographic, or inertial sensors are the most used technologies for detecting hand gestures (Cho et al., 2017; Ghafoor et al., 2017; Bisi et al., 2018; Polfreman, 2018). Visual-based hand gesture recognition systems do not need any device to wear, allowing for extreme freedom of use. Such remote sensing is very attractive, but its performances are heavily influenced by many factors such as camera field of view, challenging image processing, illumination conditions, objects overlapping, etc. (Chakraborty et al., 2017; Abraham et al., 2018). Devices based on surface electromyography (sEMG or simply EMG) recordings (Geng et al., 2016; Du et al., 2017) need electrodes in steady contact with the skin, and they are prone to motion artifacts, electromagnetic noise, and crosstalk with other biopotentials. They also require real-time processing of the raw sEMG signals to extrapolate useful features (e.g., sEMG envelope/RMS) (Parajuli et al., 2019). As example, Myo Armband by Thalmic Labs^1^, a commercial device based on eight sEMG sensors and an inertial platform, allows the user to interface via Bluetooth with PCs or mobile devices to control supported applications (Nymoen et al., 2015; Sathiyanarayanan and Rajan, 2016; Myoband, 2019) including robot motion (Bisi et al., 2018).

As an alternative to sEMG, other sensors can monitor the mechanical muscular activity, and some are briefly presented below. A pressure sensors array coupled to air-bladders mounted on an armband was proposed to detect hand motion (accuracy of 90%) by monitoring the swelling of muscles (Jung et al., 2015). The air bladders are cumbersome, uncomfortable, and not widely adaptable. A wristband composed of an array of barometric pressure sensors was proposed to estimate tendons and muscle motions during gestures (Zhu et al., 2018), reaching a classification accuracy of wrist gestures of 98%. A combination of sEMG electrodes and microphones (Caramiaux et al., 2015) was used to detect both electrical muscle activity and the mechanomyogram (MMG – i.e., mechanical vibrations produced during muscle contraction). The microphones presented high sensitivity to noise and motion artifacts, in addition to the aforementioned EMG problems. A conventional ultrasound probe fixed to the forearm was proposed for finger motion recognition, proving accuracy of 96% (Huang et al., 2017). This approach resulted very cumbersome, uncomfortable, and required a complex image processing for gestures features extraction. Furthermore, piezoelectric sensors were used to estimate finger gestures (accuracy of 97%) by recording the vibrations and shape changes that occur at the wrist due to muscles and tendons motions (Booth and Goldsmith, 2018). These kinds of sensors could also be employed to harvest energy from body movements, including upper limb motion (Elahi et al., 2018).

Other recent studies (Giovanelli and Farella, 2016) presented devices for gesture recognition based on an array of force-sensitive resistors (FSRs^2^) (Interlink Electronics, 2019). A combination of two sEMG and four FSR sensors, mounted on a wrist strap, can be used to classify finger movements scoring accuracy of 96% (McIntosh et al., 2016). An armband equipped with 16 FSR sensors positioned on both wrists and forearms (Jiang et al., 2017) allowed the classification of several hand gestures with an accuracy of about 97%. A similar device equipped with eight FSR sensors, tested on amputees (Cho et al., 2016) while trying to mirror different hand grips in their residual forearm muscles, yielded an accuracy of 70%. Moreover, a high-density grid of 126 FSR sensors (Radmand et al., 2016) embedded in a forearm prosthetic socket and tested on healthy subjects to recognize arm positions, yielded an accuracy of 99.7%.

However, the approaches proposing pressure sensors wrapped around the wrist do not directly monitor muscle contraction, but rather tension of tendons. Moreover, even in the cases of FSR arrays applied on the forearm, to the best of our knowledge, the detected signals were not proven to be equivalent to EMG.

The aim of this study was to investigate the possibility to recognize hand gestures by monitoring the contractions of a reduced number of specific forearm muscles, via the bespoke FSR-based sensors, which demonstrated to provide signals quite similar to the EMG linear envelope (EMG-LE) (Esposito et al., 2018). To reach this goal, a new gesture recognition armband is presented; it is equipped with only three FSR-based sensors, applied on specific forearm muscles to recognize eight hand gestures. The armband is designed to be easily wearable and adjustable for any user. Thanks to the similarity with the EMG-LE (Esposito et al., 2018), the device could be reconfigured to resemble previous, well-established EMG-based HMIs (e.g., exergaming applications for patients during neuromotor rehabilitation) (Ma and Bechkoum, 2008).

## Materials and Methods

### Piezoresistive Array Armband Design

**FIGURE 1:**
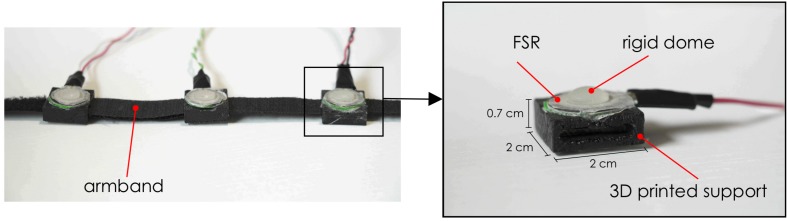
Piezoresistive array armband: **Left**, the armband with three FSRs; **Right**, an enlargement of the FSR sensor mounted on its 3D printed support with actual dimensions.

The armband consists of three piezoresistive FSRs (Interlink FSR 402) mounted on an inextensible band by means of 3D printed rigid supports (Figure 1). An FSR changes its electrical resistance in the function of the applied force (Interlink Electronics, 2019). The FSR active area is suitably mechanically coupled to the muscle through a rigid dome, which enables the measurement of muscle volume changes during contraction (Esposito et al., 2018). The support was designed with a housing site for the FSR, and an opening to allow sensor sliding along the band and precise positioning on a target muscle. The armband can be wrapped around user’s forearm and fastened with a Velcro strip in order to measure muscle contractions and recognize hand gestures. Indeed, each gesture generates a characteristic force distribution on the sensors, and this allows discriminating the intentional movements.

**FIGURE 2:**
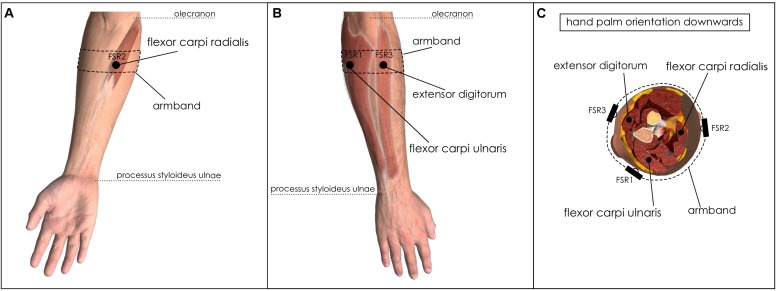
Placements of FSRs on forearm muscles. **(A)** Ventral view of right forearm: FSR2 sensor on flexor carpi radialis; **(B)** Dorsal view of the right forearm: FSR1 on flexor carpi ulnaris and FSR3 on extensor digitorum; **(C)** Right forearm cross-section: FSRs placement onto the aforementioned muscles.

Given the similarity between the FSR-based sensor output and the EMG-LE (Esposito et al., 2018), the muscle sensors should be positioned above the muscle belly as for EMG detection. The chosen muscles should be superficial to allow advantageous signal to noise ratio. Moreover, since the FSR-based sensors are embedded in an armband, the pick-up points should belong to a circumference that wraps around the forearm. Three forearm muscles were preferred to better discriminate the different hand gestures. In detail, FSR1 was applied on flexor carpi ulnaris, FSR2 on flexor carpi radialis, and FSR3 on extensor digitorum. The armband was positioned proximally at 25% of the distance between the olecranon and the process styloideus ulnae of the right forearm (Figure 2). Indeed, a functional–anatomical analysis of the forearm muscles (Drake et al., 2014) revealed that flexor carpi ulnaris is mainly involved in wrist flexion and wrist adduction; flexor carpi radialis in wrist flexion and wrist abduction; and extensor digitorum in fingers extension, fingers abduction, and wrist extension.

**FIGURE 3:**
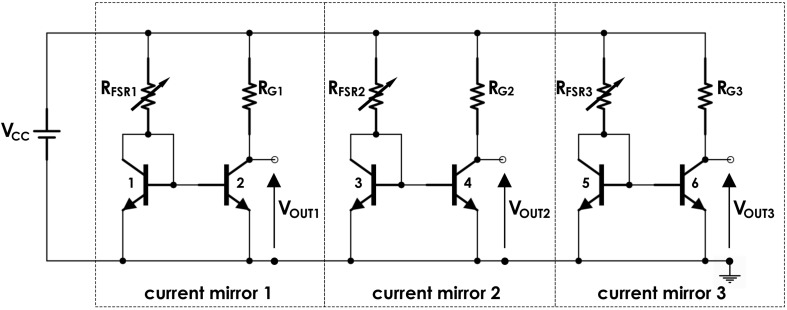
FSR sensors conditioning circuit based on mirror current circuits.

A current mirror (Figure 3) was used as a conditioning circuit for each FSR sensor (Esposito et al., 2019a, b). It was made of a pair of common npn BJT (2N2222), positioned very close to each other. Basically, the current mirror replicates the FSR sensor (*R*_FSR_) current in the gain resistor (*R*_G_), thus providing a linear load-to-voltage response and allowing the output voltage to swing through the full voltage supply range. The sensibility of each muscle sensor can be varied by changing the *R*_G_ value. Thanks to its low energy consumption, this conditioning circuit can be directly supplied by microcontrollers or ADC boards (e.g., 3.3 or 5 V). *V*_CC_ was set to 5 V, and the gain resistors *R*_G1_, *R*_G2_, and *R*_G3_ were set to 850, 790, and 960 Ω, respectively, to equalize the gains of the three channels.

Static calibrations were performed for each FSR sensor to evaluate the relationship between the muscular force exerted on the FSR, reported in kilograms, and the voltage output *V*_OUT_ (Figure 3; Esposito et al., 2018). Each sensor was placed on a precision electronic scale, then different weights were applied on active sensor area perpendicularly to the dome, and the corresponding output voltages were recorded. The output signals were acquired at 1 kHz sampling frequency with 12-bit precision by means of National Instruments NI USB-6008 acquisition board.

### Machine Learning Algorithms Applied to Hand Gesture Classification

**FIGURE 4:**
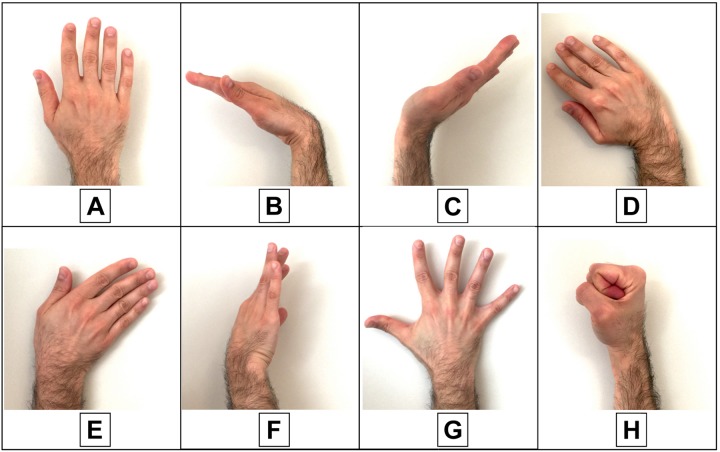
Performed hand gestures: **(A)** rest; **(B)** wrist flexion; **(C)** wrist extension; **(D)** wrist adduction; **(E)** wrist abduction; **(F)** wrist rotation (supination); **(G)** fingers abduction; **(H)** clenched fist.

The experimental tests involved 10 subjects (eight men and two women aged from 25 to 64 years), who provided their informed and written consent. Each participant comfortably sat on an adjustable height chair, leaning against its fixed seatback, in front of a desk with a computer screen. He was asked to place his elbow on the desk, forming an angle of about 45° between the forearm and the desktop. The armband was appropriately positioned on the forearm, and the pressure at rest was recorded by the sensors and resulted 100 g/cm^2^ on average. The subjects were asked to perform 10 repetitions of each hand gesture class (Figure 4) in the following order: rest; wrist flexion; wrist extension; wrist adduction; wrist abduction; wrist rotation (supination); finger abduction; clenched fist; holding the final hand posture for a couple of seconds; and resting for a few seconds before the next movement. After the 10 repetitions of each hand gesture class, the participant was allowed to rest for about a minute. Simultaneous recordings from the three FSR sensors (*V*_OUT 1–2–3_) were collected via the NI USB-6008 board at 1 kHz sampling frequency with 12-bit precision.

**FIGURE 5:**
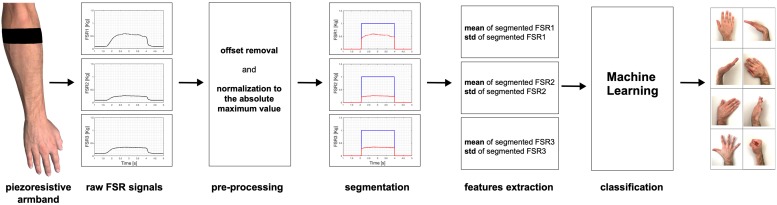
Schematic illustration of the hand gesture recognition system.

The raw signals were firstly pre-processed, by subtracting the minimum signal values recorded at rest (FSR offsets due to the armband fastening pressure) and normalizing to the absolute maximum value (Figure 5). In order to avoid manual selection of each hand gesture, pre-processed data were automatically segmented to extract the time intervals corresponding to the final hand postures. Segmentation was achieved by selecting the FSR signal with maximum variation (peak-to-peak amplitude) and applying a heuristically chosen threshold set at 40% of this value, which guaranteed appropriate segmentation of all gestures. Means and standard deviations (SDs) of the three FSR signals were computed for each segment. Then, for each gesture instance, the three means and the three SDs computed in the corresponding segment were considered as features. In detail, the features extracted from all the gestures instances in a single trial of a subject were assembled in a database consisting of an 80 × 7 matrix (10 repetitions for each of the eight hand gestures); each row corresponded to a single gesture instance and was composed by the following seven elements: (FSR1_mean, FSR2_mean, FSR3_mean, FSR1_SD, FSR2_SD, FSR3_SD, and GESTURE_LABEL).

Then, different machine learning algorithms (linear/polynomial/radial basis function-support vector machines; linear discriminant analysis; quadratic discriminant analysis; random forest; K-nearest neighbors, and neural networks) were used for model training and data classification, by means of “Weka” software (Frank et al., 2016). The conceptual scheme of the entire process of hand gestures classification is depicted in Figure 5.

Classification performances were assessed by applying the 10-fold and leave-one-out cross validations on each of the 10 subjects’ databases. In 10-fold cross-validation, the dataset is randomly divided into 10 subsets of equal size, and then each subset is tested using the classifier trained on the remaining nine subsets. Then, the obtained 10 classification accuracies were averaged to provide an overall classification accuracy. Instead, leave-one-out cross-validation is simply *n*-fold cross-validation, where *n* is the number of instances in the dataset. Each instance, in turn, is left out, and the learning method is trained on all the remaining instances. Finally, all the *n* classification accuracies were averaged to yield an overall classification accuracy (Witten et al., 2016).

Furthermore, the classification performances of the different machine learning algorithms were also tested on a combined database, obtained by joining all subjects’ databases.

Finally, the possibility to classify gestures with less than three sensors was tested by considering features from different sensors pairs (FSR1-FSR2, FSR1-FSR3, and FSR2-FSR3) and even from a single sensor (FSR1, FSR2, and FSR3). In the case of sensors pairs, each instance is characterized by four features (two means and two SDs), while for a single sensor, the features reduced to two.

### Reproducibility Test

A reproducibility test was also performed to assess the possibility to use a model trained in a previous trial to classify gestures performed in a subsequent trial. The data acquired by the 10 subjects (10 repetitions for each of the 8 gestures, as described in the section “Machine Learning Algorithms Applied to Hand Gesture Classification”) were used to construct the “linear SVM” prevision model for each subject. Then, in a subsequent trial, the same subjects wore again the device and performed a randomized gestures sequence guided by a video. The video showed a sequence of icons representing the gestures to be performed (50 randomly chosen gestures separated by the rest condition). For each subject, the data collected in this last trial were classified using the model obtained from the previous trial. The entire procedure for the reproducibility test was repeated using an LDA classifier.

### Real-Time Implementation of Hand Gesture Recognition

**FIGURE 6:**
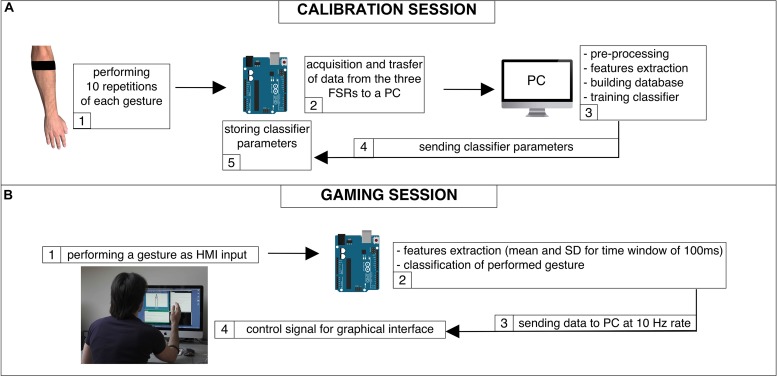
Real-time HMI: **(A)** block diagram of the calibration phase; **(B)** block diagram of the gaming session.

A linear SVM classifier was implemented on an Arduino UNO board^3^ (D’Ausilio, 2012; Arduino, 2019), equipped with an ATmega328 (Atmel) microcontroller, to provide real-time gesture recognition. The three outputs of the FSR sensors conditioning circuit were directly connected to the analog inputs of the board. In addition, custom graphical user interfaces (GUI) were designed by means of “Processing” software^4^ (Processing, 2019) to facilitate interactive armband calibration and to allow real-time user interaction with a computer. The real-time application involved the steps described below. The subject was asked to wear the armband and to perform the same sequence of gestures described in the section “Machine Learning Algorithms Applied to Hand Gesture Classification,” for device calibration. Data were sent to the PC and used to train a linear SVM classifier by means of Weka software; the trained classifier parameters were sent to the Arduino board, and the calibration phase was completed (Figure 6A). The videogame started on the PC screen and the Arduino board performed real-time classification of the current gesture: extracting gesture features (mean and SD) every 100 ms, making a classification and sending this information (coded in 1 byte) to the PC at a 10 Hz rate, via USB communication (Figure 6B). The subject started to play, and the Arduino board output was used to replace the keyboard and mouse controls. The subject never removed the armband between these steps. For each gaming session, the gestures correctly recognized in real-time were annotated and then their percentages were computed. Each user was also asked to evaluate the comfort and effectiveness of the device on a 0-to-10 scale. The implementation of a real-time LDA classifier was further tested, repeating the same procedure described for the linear SVM (Hong et al., 2018).

Moreover, in order to verify a viable real-time classification, the mean and standard deviation parameters were computed using shorter FSR signal tracts than the segmented ones (the section “Machine Learning Algorithms Applied to Hand Gesture Classification”). However, due to the stationarity properties of the FSR signal during a particular gesture, these concise statistical parameters do not differ from those computed on larger time windows and used to train the classifier.

## Results

### Signals Pre-processing and Hand Gestures Classification

**FIGURE 7:**
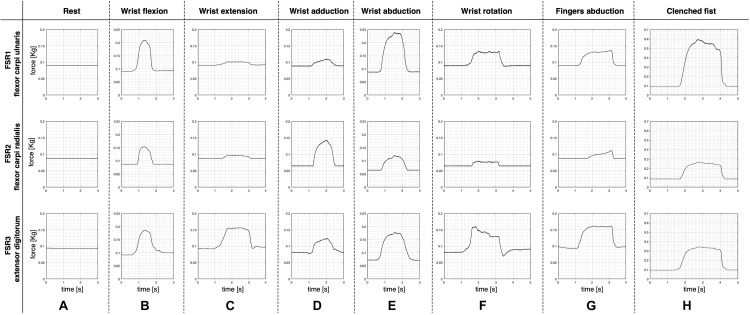
Examples of raw signals (subject #3) recorded by the three FSRs for each performed gesture: **(A)** rest; **(B)** wrist flexion, **(C)** wrist extension, **(D)** wrist adduction, **(E)** wrist abduction, **(F)** wrist rotation (supination); **(G)** fingers abduction; **(H)** clenched fist. Signal amplitudes are expressed in kilograms and different force scales were used.

Figure 7 shows an example of the FSRs raw signals for each performed hand gesture (subject #3). Different intensity force scales were used to better appreciate the signals shapes.

**FIGURE 8:**
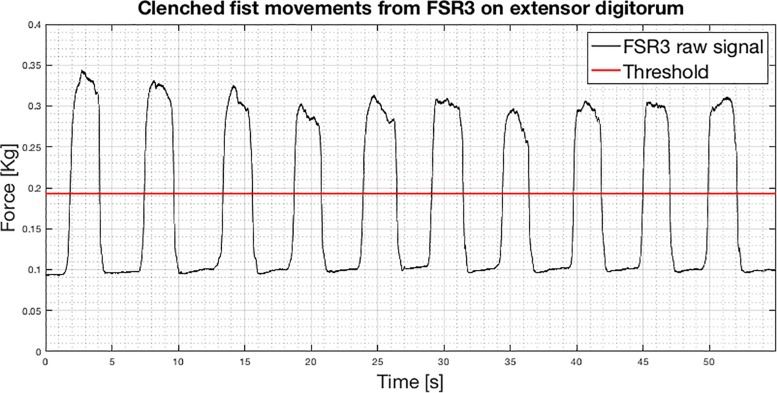
Recording of 10 consecutive clenched fist movements from FSR3 (subject #3): FSR3 raw signal with the superimposed threshold (red line).

An example of raw signal segmentation is showed in Figure 8. The segmentation function was achieved by applying a threshold set at 40% of the FSR3 maximum signal variation. The segmentation allowed us to extract only the samples associated with the fully reached gesture while discarding the initial and final transients.

**FIGURE 9:**
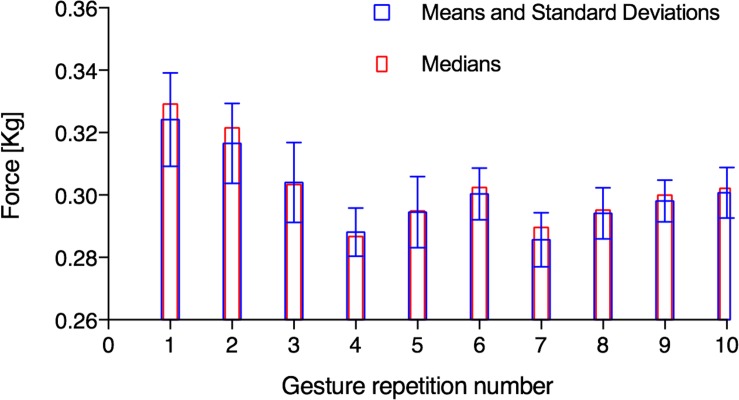
Means, standard deviations, and medians related to the segmented FSR3 signals of 10 clenched fist movements showed in Figure 8.

Moreover, analyzing the values of the segmented signals for each clenched fist movement in Figure 8, it was found that the distributions of the occurrences do not seem Gaussian. These probability distributions showed up also from the segmented signals related to the other gestures. The median, as an alternative to the mean, would be another possible feature. As an example, Figure 9 shows the means, the standard deviations, and the medians referred to the segmented signals depicted in Figure 8. In this case, the percentage variation between the mean and the median was <2% for each repetition. Comparable percentages were also found in the segmented signals related to the other gestures. Hence, there is not practical convenience in using medians instead of means because it would increase the computational burden (critical for real-time applications).

**FIGURE 10:**
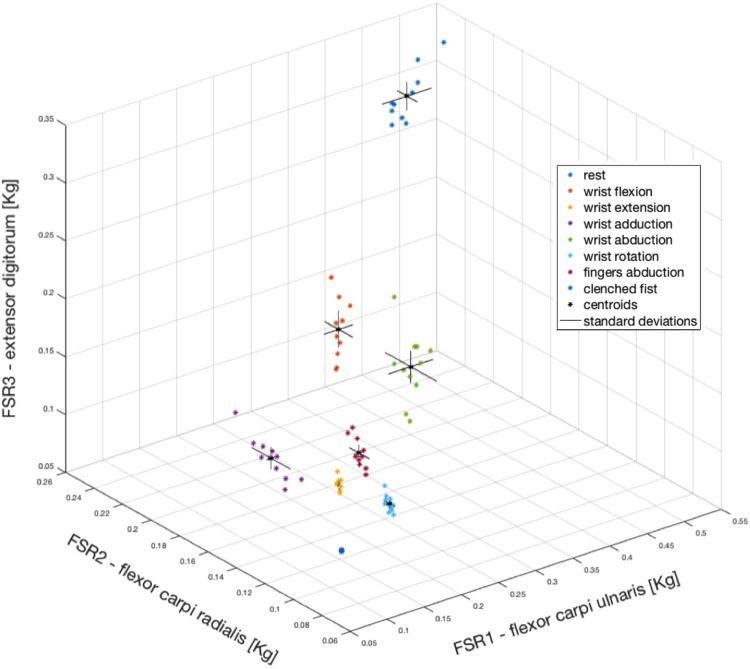
Mean values computed for each of the 10 repetitions of each gesture (coded with different colors). FSR1, FSR2, FSR3 correspond to *x*, *y*, *z* axes, respectively. For each gesture, the centroid is depicted as a black asterisk and the standard deviations in the three directions as continuous black lines.

As an example, Figure 10 shows the means corresponding to the 10 repetitions of each gesture (subject #3) with different colors (see legend of Figure 10) in a three-dimensional space (*x*, *y*, and *z* axes correspond to FSR1, FSR2, and FSR3, respectively). In addition, data were enriched by reporting centroids and standard deviations (computed in the three directions). Gestures appeared to be confined in specific regions, which did not overlap with each other. It is interesting to note that the rest condition was located around a point that represented the grip force of the armband (here about 0.1 kg).

Considering all three FSRs, the classification accuracy achieved for each subject, by means of the different algorithms and cross-validation methods, are shown in Table 1.

**TABLE 1:** Classification accuracies (in percentage) on 10 different subjects, using different machine learning algorithms [linear SVM (L-SVM), polynomial SVM (P-SVM), radial basis function SVM (RBF-SVM), linear discriminant analysis (LDA), quadratic discriminant analysis (QDA), random forest (RF), K-nearest neighbors (K-NN), and neural networks (NN)] and different cross-validation methods [10-fold (CV1) and leave-one-out (CV2)].

	**L-SVM**		**P-SVM**		**RBF-SVM**		**LDA**		**QDA**		**RF**		**KNN**		**NN**	
	**CV1**	**CV2**	**CV1**	**CV2**	**CV1**	**CV2**	**CV1**	**CV2**	**CV1**	**CV2**	**CV1**	**CV2**	**CV1**	**CV2**	**CV1**	**CV2**
S1	95	95	87.5	81.25	93.25	91.25	97.5	97.5	97.5	97.5	96.25	96.25	91.25	91.25	90	92.5
S2	92.5	87.5	73.75	76.25	90	88.75	95	96.25	96.25	96.25	89	88.75	88.50	88.75	83.75	86.25
S3	98.75	98.75	82.5	78.75	97.5	96.25	97.5	96.25	100	100	97.5	97.5	95	93.75	97	96.25
S4	96.25	96.25	80	83.75	96.25	96.25	96.25	96.25	98.75	98.75	97.5	97.5	100	100	100	100
S5	90	88.75	75	73.75	90	97.5	93.75	92.5	97.5	97.5	93.5	93.75	91	90	91.5	90
S6	100	100	85	86.25	100	100	100	100	98.75	98.75	97.5	97.5	98.75	98.75	98.75	98.75
S7	97.5	97.5	97.5	93.75	97.5	97.5	100	100	98.75	98.75	97.5	95	100	100	98.75	97.5
S8	97.5	96.25	92.5	92.5	97.5	96.25	98.75	98.75	98.75	98.75	100	100	97.5	97.5	98.75	98.75
S9	96.25	96.25	83.75	86.25	97.5	96.25	96.25	96.25	98.75	98.75	98.75	98.75	98.75	98.75	100	100
S10	96.25	95	75	86.25	96.25	96.25	97.5	96.25	93.75	93.75	93.75	93.75	98.75	98.75	97.5	97.5

**FIGURE 11:**
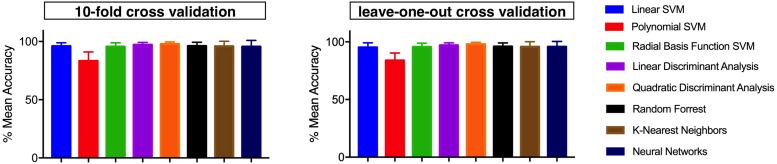
Means and standard deviations of the accuracies achieved across the 10 participants, by means of the different machine learning algorithms and for each tested cross-validation method (**Left**, 10-fold and **Right**, leave-one-out).

Figure 11 shows the means and the standard deviations of the accuracies achieved across all participants, using the aforementioned machine learning algorithms and the two cross-validation methods.

Table 1 shows that linear SVM and LDA algorithms allow to obtain higher classification accuracies with lower computational complexities, compared to all the other evaluated machine learning algorithms. Therefore, more extended analysis was focused on these classifiers, considering the 10-fold cross-validation.

Table 2 summarizes the classification performances achieved by considering all sensors combinations, reporting means and standard deviations of the related accuracies (across all participants). Using a single sensor, the mean classification accuracy was about 77% for linear SVM, while about 82% for LDA. Moreover, using two sensors the accuracy increased to about 91% for linear SVM, while about 92% for LDA.

**TABLE 2:** Means and standard deviations of classification accuracies (across all participants) by using linear SVM and LDA algorithms for all sensors combinations.

**Selected sensor/s**	**Linear SVM mean (SD) accuracy (%)**	**LDA mean (SD) accuracy (%)**
FSR1	80.25 (9.89)	80.62 (8.00)
FSR2	76 (9.12)	82.37 (10.38)
FSR3	73.88 (13.25)	82.05 (8.58)
FSR1 and FSR2	91.75 (8.70)	92.27 (6.96)
FSR1 and FSR3	92.25 (5.26)	91.62 (6.18)
FSR2 and FSR3	90.38 (5.68)	92.87 (4.41)
FSR1 and FSR2 and FSR3	96 (2.93)	97.25 (2.02)

Table 3 outlines the classification performances obtained for the combined database (all subjects) by using linear SVM and LDA for all sensors combinations.

**TABLE 3:** Classification accuracies reached on the combined database by using linear SVM and LDA for all sensor combinations.

**Selected sensor/s**	**Linear SVM accuracy (%)**	**LDA accuracy (%)**
FSR1	41.5	32.62
FSR2	38	28.5
FSR3	37.1	33.87
FSR1 and FSR2	49.6	44
FSR1 and FSR3	49.6	37.37
FSR2 and FSR3	51.9	41.12
FSR1 and FSR2 and FSR3	58.5	44.50

**FIGURE 12:**
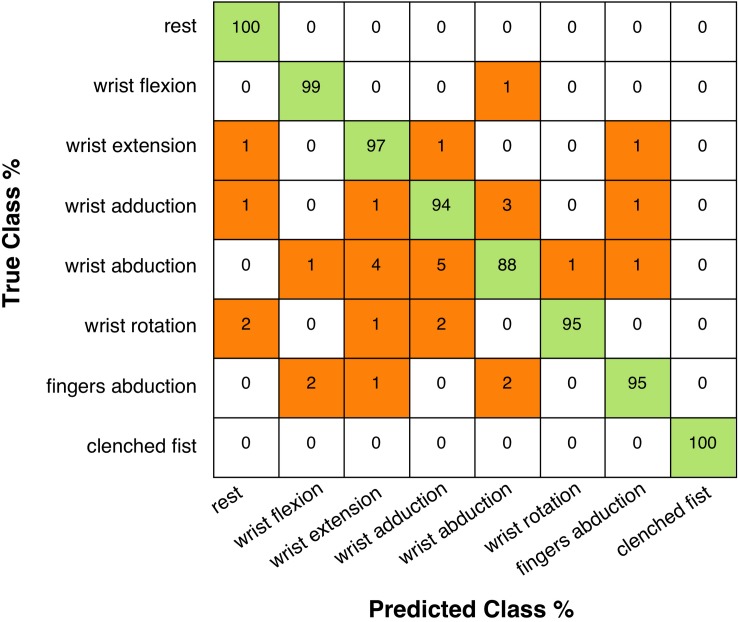
Confusion matrix (across all participants) presenting the linear SVM classification accuracies (in percentages): rows correspond to true performed hand gestures and columns to predicted hand gestures.

Table 4 shows the classification accuracies reached with linear SVM, for each subject and hand gesture class. The average accuracy across all participants resulted 96% (SD: 2.93%), and the confusion matrix (right and wrong average recognition percentages across all 10 subjects) is shown in Figure 12.

**TABLE 4:** Linear SVM classification accuracies (in percentage) on 10 different subjects in recognizing eight hand gestures (classes).

**Gesture (class)**	**S1**	**S2**	**S3**	**S4**	**S5**	**S6**	**S7**	**S8**	**S9**	**S10**
Rest	100	100	100	100	100	100	100	100	100	100
Wrist flexion	90	100	100	100	100	100	100	100	100	100
Wrist extension	100	90	100	100	90	100	100	100	100	90
Wrist adduction	100	90	100	100	100	100	90	90	90	80
Wrist abduction	80	70	100	80	60	100	100	90	100	100
Wrist rotation	90	100	90	100	80	100	90	100	100	100
Fingers abduction	100	90	100	90	90	100	100	100	80	100
Clenched fist	100	100	100	100	100	100	100	100	100	100

**FIGURE 13:**
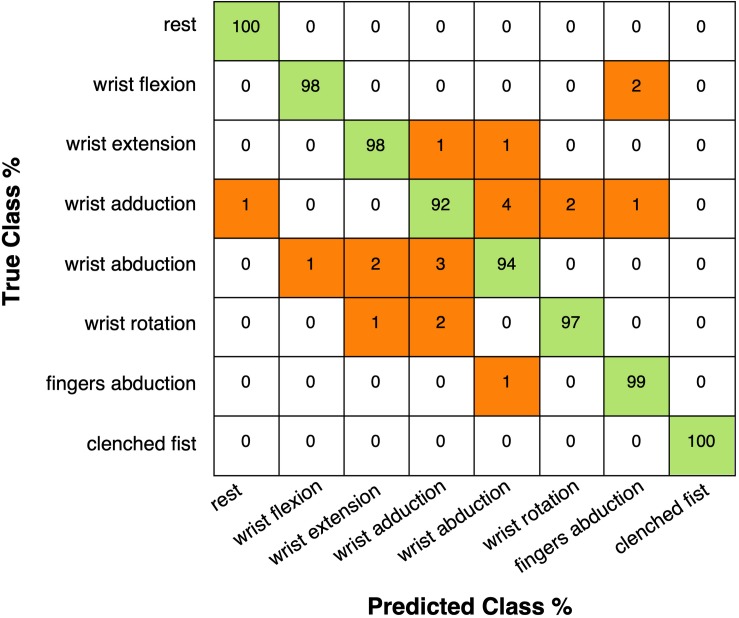
Confusion matrix (across all participants) presenting the LDA classification accuracies (in percentages): rows correspond to true performed hand gestures and columns to predicted hand gestures.

Table 5 shows the classification accuracies reached with LDA, for each subject and hand gesture class. The average accuracy across all participants resulted 97.25% (SD: 2.02%), and the confusion matrix (right and wrong average recognition percentages across all 10 subjects) is shown in Figure 13.

**TABLE 5:** LDA classification accuracies (in percentage) on 10 different subjects in recognizing eight hand gestures (classes).

**Gesture (class)**	**S1**	**S2**	**S3**	**S4**	**S5**	**S6**	**S7**	**S8**	**S9**	**S10**
Rest	100	100	100	100	100	100	100	100	100	100
Wrist flexion	100	100	100	100	90	100	100	100	100	90
Wrist extension	100	90	100	100	90	100	100	100	100	100
Wrist adduction	100	100	90	80	90	100	100	90	80	90
Wrist abduction	90	70	100	90	90	100	100	100	100	100
Wrist rotation	90	100	90	100	90	100	100	100	100	100
Fingers abduction	100	100	100	100	100	100	100	100	90	100
Clenched fist	100	100	100	100	100	100	100	100	100	100

### Reproducibility Test

During the reproducibility test, the mean classification accuracy (across all users) was 78.8% with linear SVM, while 60.25% with LDA.

### Graphical Interfaces for Practical HMI Applications

The custom graphical interface that displays icons corresponding to the recognized hand gestures was used both for calibration purposes and for quick assessment of real-time classifier performances (Figure 6). The real-time gesture recognition system was used to play various games (e.g., “Pong” videogame^5^) by replacing the mouse and keyboards commands with those provided by the Arduino board (Pong-Game, 2019). The average percentage (across all users) of correctly recognized gestures resulted 93% with linear SVM, while 90% with LDA. Subjects reported that this HMI was comfortable to wear and intuitive to use, not requiring long training to achieve good results. The mean “comfort score” was 8.3/10. The “effectiveness score” was 8.1/10 for linear SVM, and 7.8/10 for LDA.

## Discussion and Conclusion

A novel piezoresistive array armband for hand gesture recognition was presented. It was based on a reduced number of muscle contraction sensors, appropriately positioned on specific forearm muscles. Nevertheless, it allowed discriminating eight classes of hand gestures with remarkable accuracy, regardless of the specific classifier (Table 1). Classifiers based on linear SVM and LDA have low computational complexities and can be easily implemented in hardware. Therefore, more extended analysis was focused on these classifiers. The average classification accuracy across all subjects, resulted 96% for linear SVM and 97.25% for LDA. These performances were achieved by separately considering the databases associated with each user and averaging the accuracies. Instead, considering the combined database (all subjects) the linear SVM classification achieved a maximum accuracy of 58.5%, while LDA scored 44.5%. A significant classification accuracy was also achieved by considering combinations of only two sensors: the mean accuracy resulted 91.46% for linear SVM and 92.25% for LDA. As expected, the use of a single sensor led to a significant reduction in mean classification accuracy (about 77% for linear SVM and 82% for LDA). With regard to the reproducibility test (described in the section “Reproducibility Test”), the mean classification accuracy (across all subjects) was 78.8% for linear SVM and 60.25% for LDA. This reduction in accuracy suggests that each time the device is used, a new calibration (i.e., classifier training) is advisable for optimal performances. It could be interesting to extend this study to a much larger cohort of subjects, in order to obtain more reliable classification results, and also to investigate the possibility to discover common muscle activation strategies, to identify pathological behaviors, etc.

The proposed armband is extremely lightweight, simple to wear, and easily adjustable for any user. It is comfortable and unobtrusive, as proved by the low grip force values recorded at rest, and it allows to simultaneously monitor the contractions of multiple specific forearm muscles. It is also scalable in the number of sensors, thus giving the opportunity to avoid their precise positioning onto specific muscles (e.g., full sensors covered armband could be used). The extreme simplicity of FSR sensors and their conditioning circuits, along with the straightforward usability of the output signals (no additional processing required), allow to easily implement this system on low-performing, commercial platforms, also with wireless capabilities (Gargiulo et al., 2010; Bifulco et al., 2011).

The proposed HMI could be applied in “exergaming” applications: graphical interfaces can provide patients with real-time feedback on the quality of the performed gestures, inducing self-corrections of their movements. Moreover, the possibility to monitor the contractions of specific muscles would provide additional clinical information about patients’ progress. Thus, the exergaming could be used in clinical practice to make neuromotor rehabilitation processes more stimulating and enjoyable (Ordnung et al., 2017).

The encouraging results obtained with few sensors suggest the possibility to adopt this HMI also in hand prosthesis control (Polisiero et al., 2013; Bifulco et al., 2017; Sreenivasan et al., 2018), thanks to the similarity of the FSR-based sensors outputs and the EMG-LE. Indeed, the small size and flatness of the sensors make it possible to embed them inside the prosthesis socket. More generally, the muscle contraction sensors could be potentially adapted to monitor other muscles (e.g., muscles of arms, legs, shoulders, etc.), allowing them to develop a wide range of EMG-based HMI applications.

## Data Availability Statement

The datasets generated for this study are available on request to the corresponding author.

## Ethics Statement

The studies involving human participants were reviewed and approved by the Department of Electrical Engineering and Information Technologies, University Naples of Federico II, Napoli, Italy. The patients/participants provided their written informed consent to participate in this study.

## Author Contributions

DE, EA, and PB: conceptualization and methodology. DE and EA: software and investigation. AF, GG, and GD: validation. GD: resources. DE: writing – original draft preparation. EA, PB, AF, GG, and GN: writing, review, and editing. PB: supervision.

## Conflict of Interest

The authors declare that the research was conducted in the absence of any commercial or financial relationships that could be construed as a potential conflict of interest.

## Footnotes

1 https://support.getmyo.com

2 https://www.interlinkelectronics.com/request-data-sheets

3 https://store.arduino.cc/arduino-uno-rev3

4 https://processing.org

5 https://it.wikipedia.org/wiki/pong
